# Intramuscular myxoma of the biceps brachii muscle: a case report

**DOI:** 10.1093/jscr/rjac145

**Published:** 2022-04-11

**Authors:** Lina Pankratjevaite, Louise Fischer Christensen, Tadas Pranckevicius, Sigita Razbadauskiene

**Affiliations:** Department of Breast Surgery, Rigshospitalet, Copenhagen University Hospital, Copenhagen, Denmark; Department of Breast Surgery, Herlev and Gentofte Hospital, Copenhagen University Hospital, Herlev, Denmark; Department of Ophthalmology, Aalborg University Hospital, Aalborg, Denmark; Department of Surgery, Lithuanian University of Health Sciences Kaunas Hospital, Kaunas, Lithuania; Department of Pathology, Lithuanian University of Health Sciences Kaunas Hospital, Kaunas, Lithuania

**Keywords:** myxoma, intramuscular, biceps brachii, tumour, benign

## Abstract

Intramuscular myxoma (IM) is a rare benign tumour. It may occur at any age but most commonly occurs among older women. The preoperative diagnosis of IM is complicated. The diagnosis can only be definitively established by histopathological examination. The treatment of choice is radical surgical excision. We report a case of a 41-year-old woman with an IM of the biceps brachii muscle.

## INTRODUCTION

Intramuscular myxoma (IM) is a rare, benign soft tissue tumour [1–3]. Usually, these types of tumours affect the muscles of the thigh, arm, buttock and shoulder [1, 2, 4]. The tumour shows a slight female predilection and a peak incidence between the fourth and eight decades of life [5]. Usually, an IM presents as a solitary lesion [6], but in up to 5% of cases it might occur as multiple tumours [5]. Patients generally complain of a slow-growing, palpable, painless mass [4–6]. A definitive diagnosis of IM can only be made by histopathological examination of the lesion. Radical surgical excision is the treatment of choice.

In line with the SCARE (Consensus-based Surgical Case Report Guideline) criteria, we report a case of a 41-year-old woman with an IM of the biceps brachii muscle [7].

## CASE REPORT

A 41-year-old female presented with a 3-year history of a slowly growing lump on the anterior side of the left upper arm. Her past medical history was significant for type 2 diabetes mellitus, diagnosed ~10 years prior to the presentation. Otherwise, the medical history was unremarkable.

During physical examination, a 2 cm hard, non-tender lump was found in the distal part of the left biceps brachii muscle. The examination revealed no motor or sensory deficits distal to the site of the lesion, and there were no restrictions in the movement of the arm as compared with the unaffected side. Blood laboratory findings were within normal limits.

**Figure 1:**
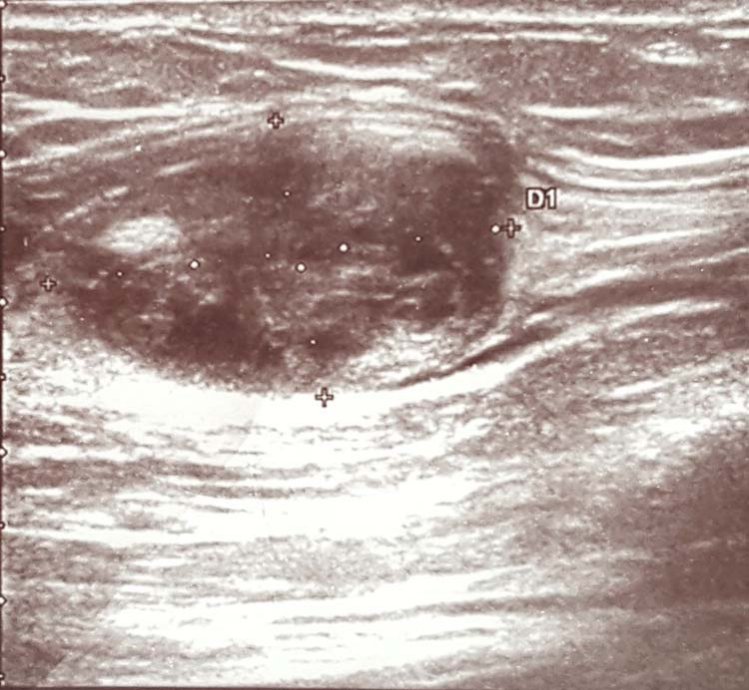
Ultrasound showing a heteroechogenic mass in the left arm.

An ultrasound scan revealed a 2.0 × 2.0 cm well-defined heteroechogenic lesion with a cystic component in the left biceps brachii muscle (Fig. 1).

**Figure 2:**
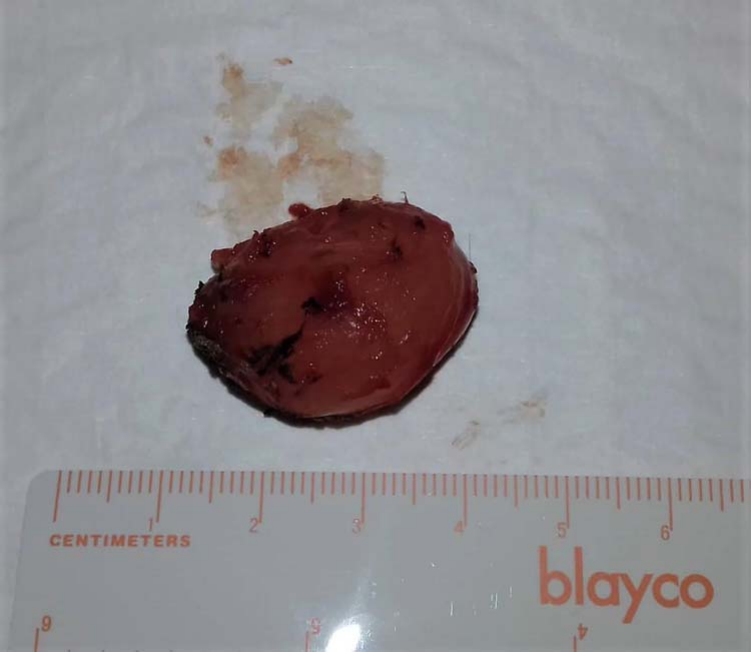
Macroscopic picture of the lesion.

The patient underwent local with clear margins surgical excision of the lesion. During surgery, the lesion appeared as a solid, well-circumscribed, encapsulated mass, and it was easily removed (Fig. 2).

**Figure 3:**
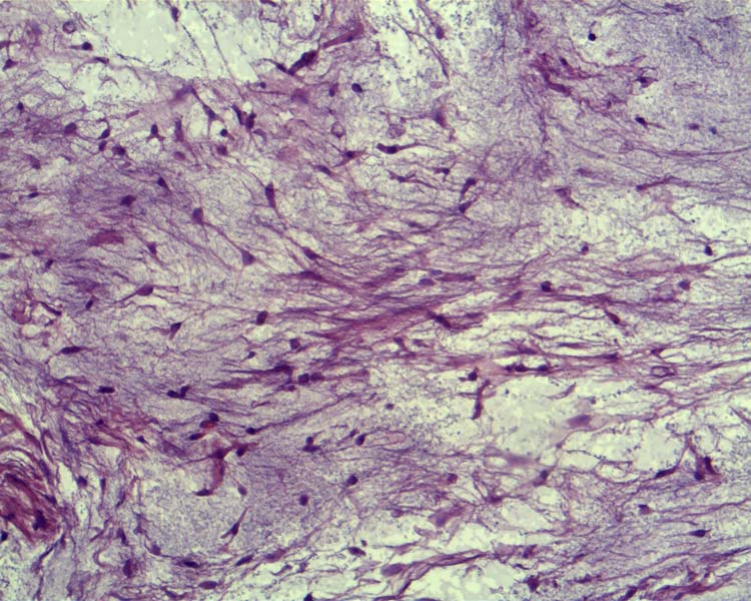
Tumour cells are small, spindle to stellate shaped with a pale indistinct cytoplasm and small hyperchromatic nuclei in which mitoses are rare (hematoxylin and eosin staining, ×20).

**Figure 4:**
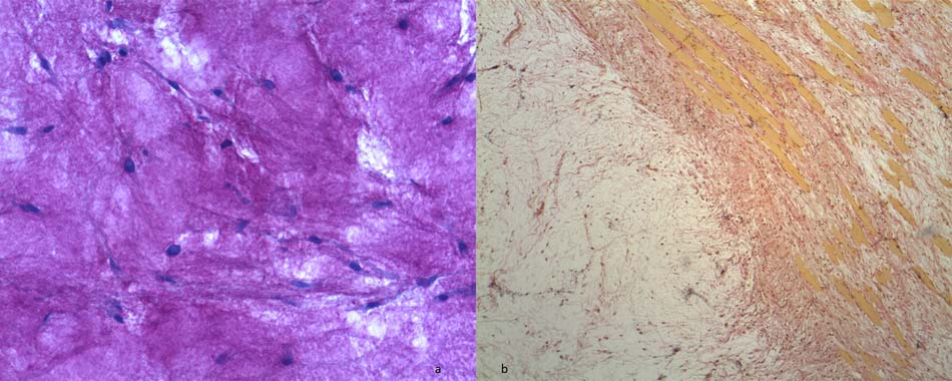
(**a**) Hypocellular myxoid tissue (periodic acid-Schiff staining, ×20). (**b**) The tumour contains strands of fibrocollagenous tissue (Van Gieson's staining).

Histopathological examination of the surgical material revealed the lesion to be an IM. The tumour was surrounded by muscle fibres and composed of spindle- and stellate-shaped cells with small, hyperchromatic nuclei (Figs 3 and 4).

The patient’s postoperative course was uneventful. She did not require any adjuvant treatment. There was no recurrence after 1 year of follow-up.

## DISCUSSION

The IM is a rare tumour with an incidence of approximately one per million [8]. It may occur within any skeletal musculature, but there is a predilection for the large, proximal groups of the extremities. While approximately half of all incidences occur in the thigh musculature, it has previously been estimated that <10% occur in the upper arm [9].

The aetiology of the IM is still unknown [6], although there is evidence that it may be associated with mutations in the *GNAS* (guanine nucleotide binding protein, alpha stimulating) gene [5, 10]. The tumour may present as a solitary mass [6] or very rarely as multiple lesions [5]. Single or usually multiple IMs may occur in conjunction with fibrous dysplasia, known as Mazabraud’s syndrome [11], and it has therefore been recommended to routinely examine patients who are diagnosed with an IM for the presence of fibrous dysplasia [1].

It is difficult to obtain a definitive IM diagnosis through non-invasive diagnostic methods, as the tumour presents itself with non-specific clinical symptoms and is easily mistaken for other tumours in imaging studies.

Clinically, an IM most frequently manifests itself as a slow-growing, palpable, painless mass [4–6], as was the case with the patient presented in this report.

Imaging techniques may include X-ray, ultrasound, computed tomography (CT scan) or magnetic resonance imaging (MRI). X-ray examinations may be normal or show a non-specific intramuscular mass that rarely contains calcifications [4, 12]. On ultrasound images, an IM typically appears as a well-circumscribed, hypoechogenic or heteroechogenic mass, sometimes with a cystic component [1, 11]. CT examinations usually show a well-defined, homogenous, low-density lesion [1–3]. On MRI images, an IM typically presents as a hypointense signal mass on T1-weighted images and with a hyperintense signal on T2-weighted images [3]. In the case reported herein, the ultrasound examination thus showed typical findings of an IM in the form of a well-defined, heteroechogenic lesion with a cystic component. No other imaging studies were performed.

The tumour should be differentiated from other benign lesions (such as lipoma, fibroma, haemangioma, schwannoma or cystic non-neoplastic lesions) and malignant tumours (such as myxoid liposarcoma, myxoid chondrosarcoma, fibrosarcoma or mucinous adenosarcoma metastases) [3–5, 12, 13].

Although imaging techniques are essential in the diagnostic workup, a definitive IM diagnosis can only be reached through histopathological examination. Core needle biopsy might be useful for differential diagnosis prior to treatment.

Macroscopically, IMs typically appear as oval, grey-white, gelatinous masses with a firm consistency [1, 3]. Microscopically, the lesions are usually hypocellular and hypovascular [1, 5, 13] and contain spindle- and stellate-shaped cells with small, hyperchromatic nuclei that are embedded within an abundant myxoid stroma [1, 6]. Generally, mitotic activity and pleomorphism are absent [1, 5]. In our case, the histopathological results were consistent with these typical findings of IMs.

The treatment of choice for IM is radical surgical excision. Complete excision of the lesion with clear small margins of surrounding tissue is essential whenever feasible [4]. Incomplete resections may result in local recurrences [3]. Conversely, recurrence of the tumour is extremely rare after radical excision [3, 5].

IMs do not show potential for malignant transformation and do not have a tendency to metastasize [4].

## CONFLICT OF INTEREST STATEMENT

None declared.

## FUNDING

None.
